# Single-cell transcriptomics reveals distinct effector profiles of infiltrating T cells in lupus skin and kidney

**DOI:** 10.1172/jci.insight.156341

**Published:** 2022-04-22

**Authors:** Garrett S. Dunlap, Allison C. Billi, Xianying Xing, Feiyang Ma, Mitra P. Maz, Lam C. Tsoi, Rachael Wasikowski, Jeffrey B. Hodgin, Johann E. Gudjonsson, J. Michelle Kahlenberg, Deepak A. Rao

**Affiliations:** 1 Division of Rheumatology, Inflammation, and Immunity, Brigham and Women’s Hospital and Harvard Medical School, Boston, Massachusetts, USA.; 2 Department of Dermatology, University of Michigan, Ann Arbor, Michigan, USA.; 3 Department of Molecular, Cell, and Developmental Biology, UCLA, Los Angeles, California, USA.; 4 Division of Rheumatology, Department of Internal Medicine, and; 5 Department of Pathology and Internal Medicine, Division of Nephrology, University of Michigan, Ann Arbor, Michigan, USA.

**Keywords:** Autoimmunity, Adaptive immunity, Skin, T cells

## Abstract

Cutaneous lupus is commonly present in patients with systemic lupus erythematosus (SLE). T cells have been strongly suspected to contribute to the pathology of cutaneous lupus; however, our understanding of the relevant T cell phenotypes and functions remains incomplete. Here, we present a detailed single-cell RNA-Seq profile of T and NK cell populations present within lesional and nonlesional skin biopsies of patients with cutaneous lupus. T cells across clusters from lesional and nonlesional skin biopsies expressed elevated levels of IFN-simulated genes (ISGs). Compared with T cells from control skin, however, T cells from cutaneous lupus lesions did not show elevated expression profiles of activation, cytotoxicity, or exhaustion. Integrated analyses indicated that skin lymphocytes appeared less activated and lacked the expanded cytotoxic populations prominent in lupus nephritis kidney T/NK cells. Comparison of skin T cells from lupus and systemic sclerosis skin biopsies further revealed an elevated ISG signature specific to cells from lupus biopsies. Overall, these data represent the first detailed transcriptomic analysis to our knowledge of the T and NK cells in cutaneous lupus at the single-cell level and have enabled a cross-tissue comparison that highlights stark differences in composition and activation of T/NK cells in distinct tissues in lupus.

## Introduction

Systemic lupus erythematosus (SLE) is a highly heterogenous disease, with the potential to manifest an array of pathologies across the body (1). Around 70% of individuals affected by SLE have cutaneous involvement, known as cutaneous lupus erythematosus, though such manifestations can also occur without further systemic symptoms (2). To date, no therapies specifically aimed at cutaneous lupus have been approved, and the current standard of care generally relies on topical corticosteroids and calcineurin inhibitors to attempt to address symptoms (3). A deeper understanding of the cells implicated in cutaneous lupus, both with and without broader systemic involvement, will be beneficial to the development of more efficacious and targeted therapies.

T cells are suspected to play a major role in lupus pathology. Expanded populations of CD4^+^ T follicular helper (Tfh) and T peripheral helper (Tph) cells promote B cell activation and autoantibody production (4–7). Work analyzing the role of Tregs in lupus has, on the other hand, been more contentious, with conflicting results suggesting both increased and decreased presence in the disease (8–12). Single-cell transcriptomic analyses of lupus nephritis (LN) kidney biopsies have suggested a role for cytotoxic T cell subsets in the kidneys of affected patients, with populations of NK cells, cytotoxic T cells, and granzyme K^+^CD8^+^ T cells all being highly represented among lymphocytes in kidney biopsies (13). Furthermore, histologic analyses of cutaneous lupus biopsies identified heterogenous staining of granzyme B^+^ T cells in cutaneous lesions (14). The extent to which T cell infiltrates within different target tissues in lupus — for example, skin and kidney — demonstrate similar effector phenotypes remains unclear.

Advances in single-cell RNA-Seq (scRNA-Seq) technologies have allowed for the high-throughput generation and analysis of cellular states in disease and homeostasis (15, 16). To date, these tools have been applied to multiple rheumatic diseases, including lupus (17). Previous studies in lupus have produced single-cell catalogs of the cell states present in kidney biopsies of patients with LN and in PBMCs of patients with SLE (13, 18, 19). These scRNA-Seq data sets have served as a foundation for a better understanding of the cell types relevant to lupus pathology in these tissues. A single-cell transcriptomic analysis of cutaneous lupus could likewise help to reveal insights into the pathogenesis of the disease and serve as a means to compare the role of a particular cell type, such as T cells, across multiple tissues of lupus pathology.

Here, we report the first detailed transcriptomic evaluation to our knowledge of the T and NK cells in cutaneous lupus lesions at the single-cell level. With paired lesional and nonlesional skin biopsies from patients with cutaneous lupus, as well as skin biopsies from healthy donors, we defined and compared the T cell populations present across these samples. Furthermore, we employed a previously published data set of kidney biopsies in LN patients (13) to perform an integrated analysis of the T cell states present across both pathologic kidney and skin. Lastly, we performed a comparison of skin T cells between cutaneous lupus and systemic sclerosis (SSc) patients, providing a cross-disease examination of the role of T cells across different rheumatic skin pathologies (20). Combined, these analyses reveal both parallels and distinct differences between the T cells in skin and kidney in lupus, suggesting that the effects of therapeutic targeting of T cells may differ in different target tissues.

## Results

### Single-cell transcriptomic identities of skin-localized T cells in cutaneous lupus.

**Figure 1:**
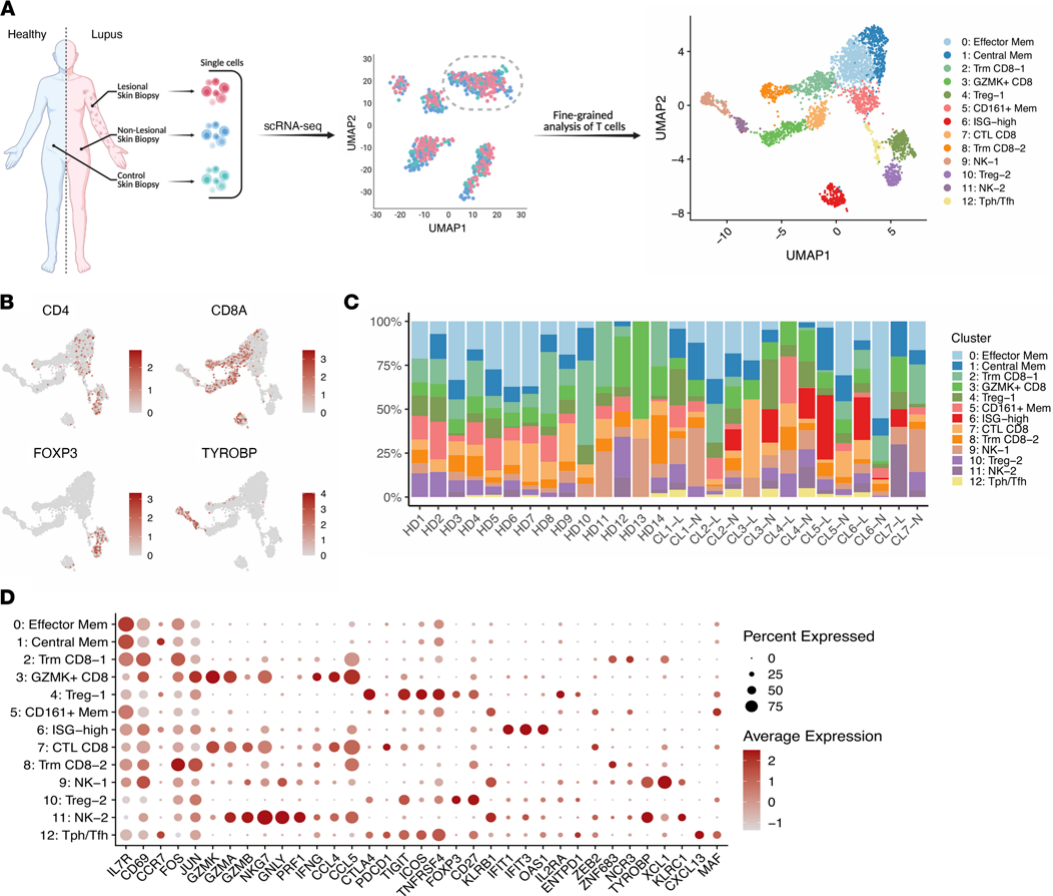
Identification of T and NK cell states in lesional and nonlesional skin biopsies of cutaneous lupus patients. (**A**) Schematic diagram of study, including sample collection, initial scRNA-Seq, and fine clustering of T cell subsets. (**B**) UMAP plots of main T cell lineage marker expression. (**C**) Bar plot of cluster assignments for captured cells in each sample. (**D**) Dot plot of differentially expressed genes in each identified cluster. HD, healthy donor; CL, cutaneous lupus; L, lesional; N, nonlesional.

Skin biopsies were obtained from both lesional and nonlesional locations on 7 patients with active cutaneous lupus, as well as 14 healthy controls. Among the cutaneous manifestations of the patients in this study, 3 had a diagnosis of discoid lupus erythematosus (DLE) and 4 had a diagnosis of subacute cutaneous lupus erythematosus (SCLE) (Supplemental Table 1; supplemental material available online with this article; https://doi.org/10.1172/jci.insight.156341DS1). Furthermore, 6 of these patients were diagnosed with SLE, while 1 had isolated cutaneous lupus. Biopsies were dissociated, and droplet-based scRNA-Seq was used to obtain the transcriptomes of T cells. Following the application of quality-control filters, we obtained a total of 3499 T and NK cells for further analysis (Figure 1A). Among these, we broadly identified populations of CD4^+^ T cells, CD8^+^ T cells, and NK cells (marked by *KLRB1* and *TYROBP* expression) (Figure 1B). Further analysis identified 13 subclusters, which largely appear independent of any batch effects (Figure 1C and Supplemental Figure 1).

We next set out to define the unique transcriptional programs of each subcluster (Supplemental Table 2). Across subclusters containing CD4^+^ cells, conventional memory T cells accounted for 3 of these clusters (clusters 0, 1, and 5). FOXP3^+^ Tregs were found across 2 clusters, both sharing expression of *CTLA4*, *ICOS*, and *CD27* (clusters 4 and 10). Notably, the Tregs associated with cluster 4 were found to have stronger expression of *CTLA4*, *TIGIT*, and *ICOS* than those in cluster 10, while those in cluster 10 were marked by higher expression of *CD27*. The differing expression profiles of these Treg clusters suggest that those cells contained in cluster 4 belong to a more activated and suppressive subset (21). In addition, we noted a population of Tfh and/or Tph cells, defined by the absence of *FOXP3* and the expression of markers such as *CXCL13*, *PDCD1*, *ICOS*, and *MAF* (Figure 1D).

Across the NK and CD8^+^ T cell clusters identified in these biopsies, tissue-resident memory CD8^+^ resident memory T cells (Trm), identified by expression of markers such as *ZNF683* and *XCL1*, formed 2 clusters (clusters 2 and 8). A population of CD8^+^ T cells marked by the strong expression of *GZMK* but relative absence of *GZMB* was identified (cluster 3), consistent with a phenotype previously recognized in LN kidneys and rheumatoid arthritis synovium (13, 22). In contrast, cluster 7 expressed higher levels of *GZMB* compared with cluster 3, suggesting a cytotoxic T lymphocyte (CTL) phenotype. Clusters 3 and 7, though, were both found to similarly express the cytokine *IFNG* and chemokines *CCL4* and *CCL5*. Aside from these CD8^+^ clusters, 2 subclusters of NK cells were identified in the data set (clusters 9 and 11). While both share a similar core transcriptional program composed of markers such as *KLRB1*, *TYROBP*, and *NKG7*, the NK cells of cluster 9 are largely differentiated by stronger expression of *XCL1*, suggestive of a CD56^bright^ NK cell population, while those of cluster 11 overexpress *PRF1*, multiple granzyme genes, and *CCL5*, likely representing a CD56^dim^ population (23).

In addition, a cluster marked by the expression of IFN-stimulated genes (ISGs) such as *IFIT1*, *IFIT3*, *IFI44*, *OAS1*, and *LY6E*, among others, was identified. This population of cells appears to be a mixture of CD4^+^, CD8^+^, and CD4^–^CD8^–^ cells, likely comprising cells from the other major subclusters (Figure 1D). This ISG-enriched cluster is consistent with previously published scRNA-Seq studies of the T cells in nephritic kidney, blood, and skin of SLE patients, further highlighting a conserved IFN signature across cell types and tissues in SLE (13, 19, 24).

### Similarly elevated IFN-response signature in T cells at both lesional and nonlesional sites in cutaneous lupus.

**Figure 2:**
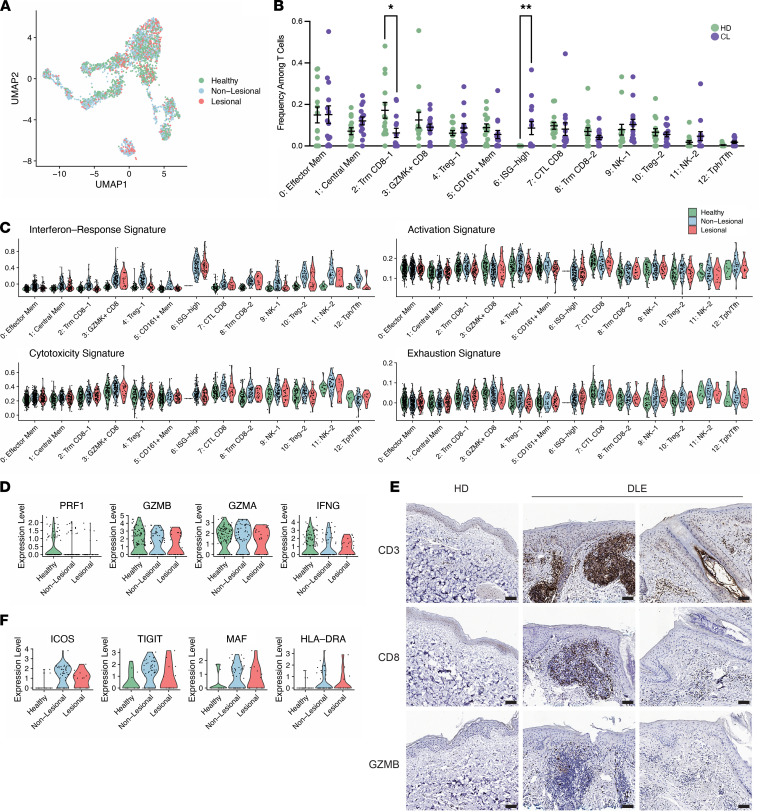
Examination of differences between lesional, nonlesional, and healthy skin biopsies. (**A**) UMAP plot of cells colored by sample type. (**B**) Comparison of the frequencies of T cells per cluster between cutaneous lupus (CL) patients and healthy donors (HD). (**C**) Violin plots of signature scores between healthy, lesional, and nonlesional cell components of each cluster. (**D**) Violin plots of the expression of select markers between healthy, lesional, and nonlesional cell components of the CTL CD8 cluster. (**E**) Representative IHC staining for CD3, CD8, and GZMB in healthy donor and discoid lupus erythematosus (DLE) skin biopsy samples. *n* = 4 biopsies used for IHC in both groups. Scale bars: 100 μM. (**F**) Violin plots of the expression of select markers between healthy, lesional, and nonlesional cell components of the Tph/Tfh cluster. Data are shown as mean ± SEM. *P < 0.05, **P < 0.01 by Kruskal-Wallis test in **B**.

We next sought to determine if certain cell subsets were differentially represented among the lesional, nonlesional, and control samples obtained by this study. Of the 3499 T and NK cells sequenced, 2116 and 1383 were collected from cutaneous lupus patients and healthy controls, respectively. In total, 687 cells were obtained from the lesional biopsies of these lupus patients, and 1429 were sequenced from their paired nonlesional samples. Accounting for differences in cell numbers for each sample type, we identified the ISG-high cluster to have greater representation in the lupus patient samples compared with healthy controls (Figure 2, A and B).

The cluster of ISG-high T cells was nearly exclusively represented by cells from the cutaneous lupus samples, including cells from both lesional and nonlesional samples (Supplemental Figure 2). In addition, an elevated ISG transcriptional signature was seen across clusters in both the lesional and nonlesional samples compared with controls (Figure 2C). This is consistent with previous studies that have identified a conserved elevation in type I IFN–regulated genes across patients with cutaneous lupus erythematosus and SLE (25–27).

We next sought to use the transcriptomic data to compare the functional status of T cells in cutaneous lupus and healthy skin biopsies. For this effort, we obtained previously identified activation-, cytotoxicity-, and exhaustion-relevant gene lists and calculated signature scores for lesional, nonlesional, and healthy controls (Supplemental Table 3). Surprisingly, T cells from cutaneous lupus lesions and nonlesional sites appeared quite similar to T cells from control skin across these measures, suggesting a lack of wide-scale activation in T cells within cutaneous lupus skin lesions (Figure 2C).

A focused analysis of the expression of key cytotoxic genes specifically within the CTL cluster similarly revealed no expression differences (Figure 2D). To evaluate the presence of cytotoxic T cells through a complementary approach, we performed IHC on healthy control and DLE skin biopsy samples. We observed CD3^+^ T cells in both healthy and DLE skin, with clusters of T cells visible in some areas of the DLE skin. A minority of the T cells were CD8^+^ cells, consistent with scRNA-Seq results. Staining for granzyme B to detect cytotoxic T cells identified a small number of granzyme B^+^ cells, while the majority of T cells did not express granzyme B (Figure 2E).

In contrast, a focused analysis on the Tph/Tfh cell cluster, a population of cells strongly implicated in pathologic T/B cell interactions in SLE, revealed some differences. Notably, we found an upregulation of costimulatory genes such as *ICOS* and *TIGIT* in Tph/Tfh cells from cutaneous lupus patients compared with their healthy control counterparts, and we further noted an upregulation of HLA-DRA and the transcription factor *MAF*, which has been demonstrated to promote Tph/Tfh cell function (Figure 2F; ref. 4).

Altogether, these data highlight the systemic nature of detection of IFN response genes in T cells of the skin and suggest a potential increase in activity of the B cell–helping Tph/Tfh cells; however, they otherwise indicate limited features of activation or cytotoxicity in skin-localized T cells in cutaneous lupus.

### Low cytotoxicity and effector signatures in T cells from cutaneous lupus compared with LN.

**Figure 3:**
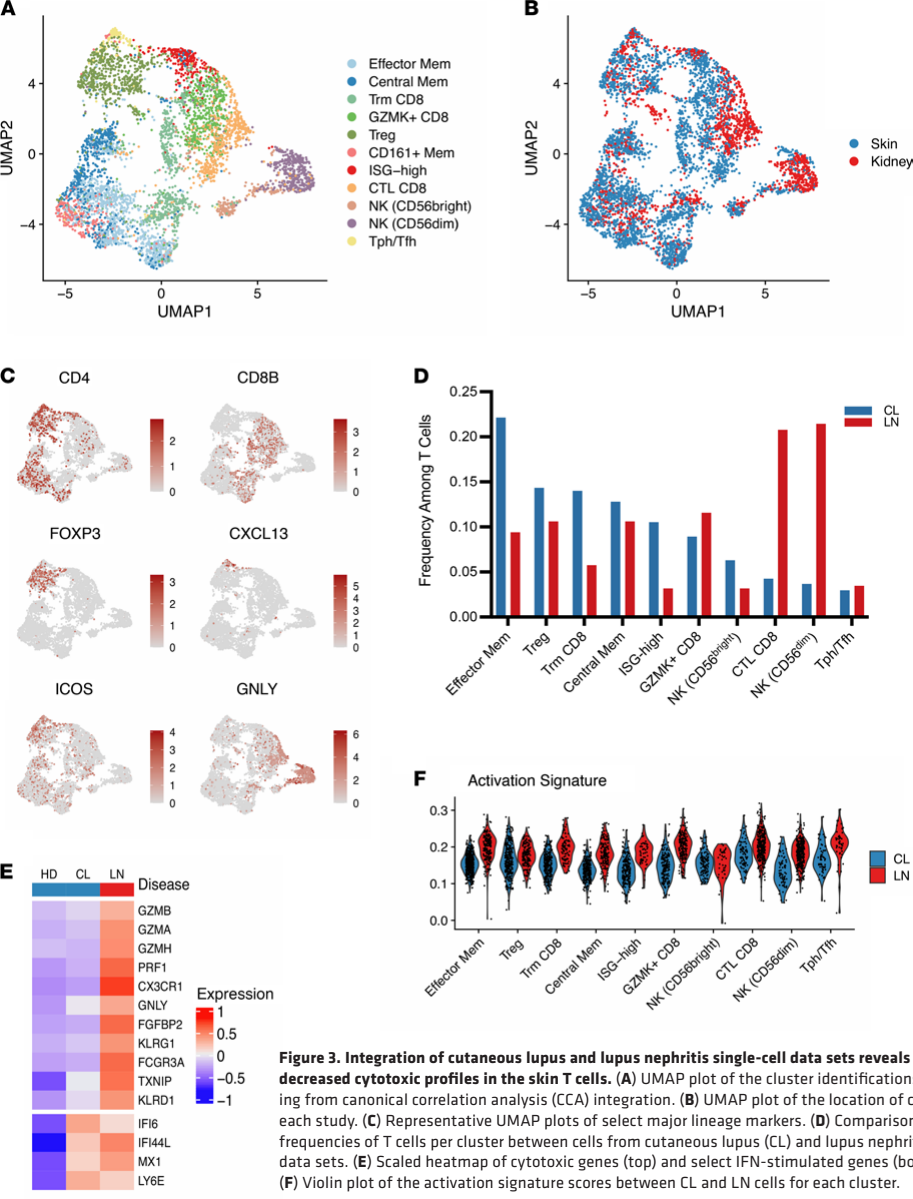
Integration of cutaneous lupus and lupus nephritis single-cell data sets reveals decreased cytotoxic profiles in the skin T cells. (**A**) UMAP plot of the cluster identifications resulting from canonical correlation analysis (CCA) integration. (**B**) UMAP plot of the location of cells from each study. (**C**) Representative UMAP plots of select major lineage markers. (**D**) Comparison of the frequencies of T cells per cluster between cells from cutaneous lupus (CL) and lupus nephritis (LN) data sets. (**E**) Scaled heatmap of cytotoxic genes (top) and select IFN-stimulated genes (bottom). (**F**) Violin plot of the activation signature scores between CL and LN cells for each cluster.

To better understand our results in the broader context of lupus, we sought to define conservation and potential differences across T cells found at different tissue sites in SLE. To accomplish this, we performed an integration of our data set with T cells from a previously published scRNA-Seq data set of immune cells obtained from kidney biopsies of patients with LN (13). Within this data set, we isolated 1719 T and NK cells from 24 patients with LN. Following integration of these data sets using canonical correlation analysis (CCA), we produced a single unified visualization of data from both tissues (Figure 3A and Supplemental Figure 3A). Cells of the same subset largely clustered together irrespective of tissue origin, and the T cell types were found in both tissues, including CD8^+^ Trm, Tregs, CD8^+^ cytotoxic T cells, Tph/Tfh cells, and NK cells (Figure 3, B and C).

When comparing representation of these cell states across tissues, we noted an increased proportion of memory T cells in the skin of cutaneous lupus patients, including CD4^+^ memory and CD8^+^ Trm subsets, along with a larger percentage of cells in the ISG-elevated cluster, when compared with kidney samples of LN patients. Conversely, we observed a strongly increased representation of cells with cytotoxic function, including CD8^+^ and NK clusters, in samples obtained from the kidneys of LN patients (Figure 3D).

Gene-level examination of cytotoxic marker expression between cutaneous lupus and LN further highlighted this difference between T cells from the different tissues, with LN T cells having increased expression of genes associated with cytotoxicity, including *GZMB*, *GZMH*, *PRF1*, and *GNLY* (Figure 3E). In comparison, ISGs showed similar expression levels between cutaneous lupus and LN T cells, indicative of the systemic IFN response in lupus. Along with an increase in cytotoxicity genes, T cells from LN kidneys also showed an increased activation signature score across clusters compared with T cells from skin (Figure 3F).

### Elevated IFN signatures in skin-localized T cells from lupus compared with SSc.

**Figure 4:**
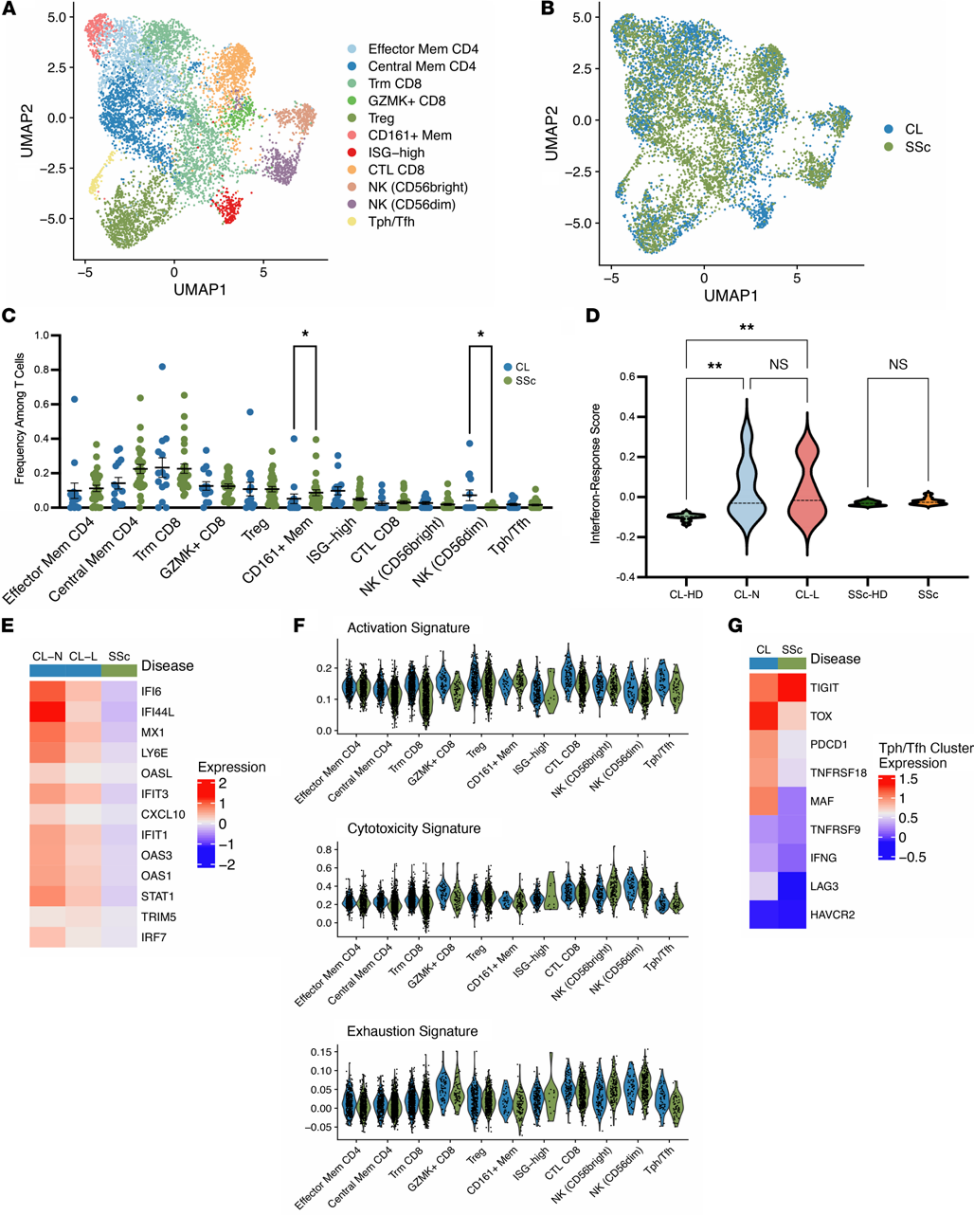
Integration of cutaneous lupus and systemic sclerosis single-cell data sets demonstrates selective ISG upregulation in lupus. (**A**) UMAP plot of the cluster identifications resulting from CCA integration. (**B**) UMAP plot of the location of cells from each study. (**C**) Comparison of the frequencies of T cells per cluster between cells from cutaneous lupus (CL) and systemic sclerosis (SSc) data sets. (**D**) Violin plot of IFN response signatures between study and sample types. (**E**) Scaled heatmap of IFN-stimulated genes. (**F**) Violin plots of signature scores between CL and SSc cells for each cluster. (**G**) Scaled heatmap of select activation and exhaustion markers in the Tph/Tfh cluster. Data are shown as mean ± SEM. *P < 0.05, **P < 0.01 by Kruskal-Wallis test in **C** and **D**.

To further extend our exploration of the T cells present in cutaneous lupus, we sought to compare our data with T cells from the skin of another rheumatic disease. A recent study focused on SSc obtained skin biopsies from 27 patients with SSc and an additional 10 healthy controls (20, 28). After isolating T cell transcripts from this data, we integrated the data set with our data set of cutaneous lupus T cells, providing a unified visualization of both sets in the same Uniform Manifold Approximation and Projection (UMAP) space (Figure 4A and Supplemental Figure 3B). Similar to our results upon integrating data from skin T cells with data from kidney T cells in lupus, we observed the presence and coclustering of all major cell types described above in T cells from both SSc skin and lupus skin samples (Figure 4B).

Comparing the distribution of cells from each disease for each cluster, we noted a deficiency of cells from SSc samples in the cluster associated with the strongest ISG signature (Figure 4C). Examination of the sample groups and healthy controls revealed that, while there is a significant increase in ISGs comparing T cells from control skin and T cells from cutaneous lupus lesional and nonlesional sites, no such difference exists between SSc samples and their respective healthy controls (Figure 4D). A heatmap analysis of the expression patterns of multiple ISGs in cutaneous lupus and SSc T cells further corroborated this finding, suggesting that IFNs more strongly influence the cutaneous T cell response in lupus than in SSc (Figure 4E).

We then aimed to profile differences in the activation and effector function of T cells between cutaneous lupus and SSc. Through an examination of signature scores, our analysis noted no differences in these scores between the cutaneous lupus and SSc components of each identified cluster (Figure 4F). Lastly, we sought to compare the Tph/Tfh cluster, which is marked by CXCL13^+^CD4^+^ T cells in cutaneous lupus and SSc. While ISGs represented many of the most upregulated genes in cutaneous lupus, we also noticed an upregulation of multiple activation and exhaustion genes. Notably, we found higher levels of *PDCD1*, *TOX*, *LAG3*, *TNFRSF18*, and *MAF* on average in T cells from cutaneous lupus samples compared with SSc samples (Figure 4G). Together, these results emphasize the strength of IFN response in lupus, even in comparison with another rheumatic disease, and suggest an increased activity of B cell–helping T cell subsets in cutaneous lupus compared with SSc.

## Discussion

This study represents the first in-depth examination of skin-localized T cells in cutaneous lupus using single-cell transcriptomics. Our analysis revealed deep transcriptional heterogeneity within the T cells collected from the skin of these patients, including subsets of CD4^+^ and CD8^+^ memory T cells, Tregs, cytotoxic T cells, Th cells, and others. One of the defining characteristics in our comparison of T cells from lupus patients and healthy donors is the significant upregulation of ISGs. Though the role and sources of IFN is increasingly well understood in the pathogenesis of lupus, including in the skin (27, 29), this study furthers the notion that systemic effects of this signaling can be detected across tissues. Our results demonstrate robust upregulation of ISGs in T cells of both the kidney and skin of lupus patients, far exceeding that in T cells from both healthy skin and skin from SSc patients.

Aside from strong differences related to IFN response, we noted surprisingly little transcriptional evidence of increased activation or effector function engagement when comparing T cells from cutaneous lupus skin with those from healthy skin. In contrast, when comparing T cells from the kidneys of lupus patients with T cells from the skin of lupus patients, our analysis suggests that the T cell infiltrates differ substantially at these 2 tissue sites. We noted a marked increase in T cells associated with cytotoxic function, and we likewise observed an increase in expression of effector function–related genes, in T cells from LN kidneys. These findings raise the possibility that T cells may play different roles in the pathology of lupus in disparate tissues, whereby T cells in organs such as the kidney may mediate cytotoxic effects that contribute to tissue injury, while T cells in the skin may contribute alternative functions, including B cell help, or may be primarily bystanders.

There are several limitations that raise caution about this interpretation of the data. First, the total number of lesional T cells analyzed is limited. Second, it is possible that T cell activation signatures are downregulated during the longer processing time required for isolation of T cells from skin. However, the robust detection of ISGs suggests that at least some of the disease-associated transcriptomic signatures are retained during processing. Third, it is possible that activated T cells are inefficiently collected or preferentially depleted during tissue processing, or that the activated cells represent a small minority of the total cells that is not well visualized in our analyses. IHC analyses support the limited number of granzyme B^+^ T cells in skin in cutaneous lupus, although additional analyses by complementary approaches will be helpful to address these considerations. It is also possible that either systemic or topical treatments may have affected the T cells studied from these patients. As treatments varied across patients, we cannot identify effects of specific treatments in this cohort. Despite these limitations, the striking differences between transcriptomes of T cells from kidney and skin appear to suggest that there may be substantial differences in their functions.

Notably, our analysis of the Tph/Tfh cells of cutaneous lupus patients suggests an increased activation of Tph/Tfh cells obtained from lesional and nonlesional lupus skin compared with control skin or SSc skin. These cells may have a role in directing B cell responses within the skin. However, the frequency of Tph/Tfh cells in cutaneous lupus lesions is low and not clearly different from controls; thus, it is also possible that these cells in skin are a reflection of the activated circulating Tph/Tfh cell populations in SLE patients (30). Furthermore, B cells were not effectively captured in this study, hindering our ability to perform deeper analyses on T and B cell interactions. The findings related to this cell subset and their potential role in the skin of lupus patients warrant further examination with larger populations of isolated cells. In addition, 6 of 7 patients in this study had SLE; thus, additional studies of patients with isolated cutaneous lupus erythematosus are needed to determine whether similar changes are observed in nonlesional skin of patients with cutaneous lupus without SLE.

The search for effective and targeted therapies for lupus remains difficult, and certain gaps in our knowledge of the diseases pathogenesis across tissues remain. Here, we delineate the phenotypes of T cells in cutaneous lupus, both in relation to healthy donor T cells as well as T cells from other affected organs. These results suggest a limited activation of T cells in the skin of lupus patients, especially in comparison with those of the kidney, and suggest that the pathologic roles of T cells in lupus differ depending on the target tissue. These findings may help to explain the differences in the efficacy of antimetabolite or T cell–directed therapies in lupus manifestations in skin and kidney, and they may influence the search for the most efficacious pathways to target to treat cutaneous lupus.

## Methods

### Subjects and sample collection

Skin punch biopsies were obtained from lesional (primarily back, chest, arms) and nonlesional (sun-protected hip) locations from 7 patients with active cutaneous lupus (Supplemental Table 1). A diagnosis of SLE was confirmed for 6 of 7 patients via the 2019 EULAR/ACR criteria (31), and the remaining patient was noted to have cutaneous involvement only. In addition, 14 healthy donors were recruited to obtain control skin punch biopsies from sun-protected skin.

### IHC

Paraffin-embedded tissue sections (DLE and control) were heated at 60°C for 30 minutes, deparaffinized, and rehydrated. Slides were placed in antigen retrieval buffer and heated at 125°C for 30 seconds in a pressure cooker water bath. After cooling, slides were treated with 3% H_2_O_2_ for 5 minutes and blocked using 10% goat serum for 30 minutes. Overnight incubation (4°C) was then performed using anti–human CD3 (Thermo Fisher Scientific, UM500048, 1:300), CD8 (Thermo Fisher Scientific, MA5-14548, 1:100), or GZMB (Abcam, ab243879, 1:200). Slides were then washed and treated with secondary antibody for 30 minutes, with peroxidase for 30 minutes, and with diaminobenzidine substrate for 2–5 minutes. Counterstain was then performed with hematoxylin and dehydrated, and then slides were mounted.

### Single-cell cDNA and library preparation

The collected biopsies were incubated overnight in 0.4% dispase (Invitrogen) in HBSS (Thermo Fisher Scientific) at 4°C as previously detailed (24). Single-cell libraries for all samples were generated using the 10× Genomics Chromium Single Cell 3′ (v3 Chemistry) protocol through the University of Michigan Genomics Core. Finally, libraries were sequenced on an Illumina NovaSeq 6000 sequencer to generate 150 bp paired end reads.

### Data analysis

#### Processing FASTQ reads.

Initial processing of the sequencing output, including quality control, read alignment to a reference genome (GRCh38), and gene quantification was performed using the 10× Genomics Cell Ranger pipeline (v3.1.0). Following the generation of barcode and UMI counts, samples were merged into a single expression matrix using the cellranger aggr pipeline.

#### Data subsetting and quality control filtering.

T and NK cell clusters were subset from the above data set of skin biopsies from cutaneous lupus patients and healthy donors in Seurat (v4.0.3). The remaining cells were then filtered to only include cells with between 200 and 3500 detected features and those with less than 15% of reads associated with mitochondrial genes.

#### Dimensionality reduction and clustering.

Cells that passed all subset and filter steps then underwent another round of principal component analysis, where the first 20 PCAs were subsequently used for downstream analysis. To ensure integration of our samples, we corrected batch effects at the level of the sample using Harmony (32). We employed Harmony using our PCA reduction with theta = 2, max.iter.cluster = 20, and max.iter.harmony = 10. After integration, cells were clustered using Louvain clustering in Seurat with a resolution of 0.6, determined through an iterative analysis of clustering results. Resulting clusters were then visualized using UMAP plots. Differential gene expression between clusters was determined using a Wilcoxon rank-sum test, and the resultant list was filtered to only include genes with a log fold-change greater than 0.25 and those that were detected in at least 50% of the population being tested.

#### Data set integration.

Inter–data set integration was performed using CCA in Seurat (33). First, the LN and SSc data sets were downloaded and filtered on the above quality control metrics, before being clustered. The T and NK cells were then subset out from the respective data sets. Following this, CCA integration was performed by first finding anchors between the data set pairs, using the first 20 dimensions. Afterward, the *IntegrateData* function in Seurat was used to display the data sets on the same UMAP.

## Data availability

The cutaneous lupus single-cell transcriptomics data analyzed in this publication is available in the Gene Expression Omnibus (GEO) database under the accession no. GSE186476.

The LN analyzed during this study can be accessed within the Single Cell Portal hosted by the Broad Institute using the study ID SCP279.

The SSc data analyzed during this study is available in the GEO database under the accession nos. GSE138669 and GSE181957.

## Statistics

Statistical analysis was performed using Kruskal-Wallis testing (Figures 2 and 4) in GraphPad Prism (v9). For all statistical tests, *P < 0.05 and **P < 0.01.

## Study approval

Sample collection for this study was approved by the University of Michigan IRB and conducted according to the principles of the Declaration of Helsinki.

## Author contributions

GSD conceived and designed the analysis, analyzed data, and wrote the manuscript. ACB, MPM, RW, LCT, and JBH collected and processed samples. XX completed tissue staining and image collection. JEG collected samples and completed tissue staining and image collection. FM analyzed data. JMK conceived and designed the analysis and supervised the work. DAR conceived and designed the analysis, analyzed data, wrote the manuscript, and supervised the work.

## Supplementary Material

Supplemental data

Supplemental table 1

Supplemental table 2

Supplemental table 3

## Version 1 03/15/2022

In-Press Preview

## Version 2 04/22/2022

Electronic publication
