# Supplementary material for: Single-cell transcriptomics reveals distinct effector profiles of infiltrating T cells in lupus skin and kidney
Source: JCI Insight. 2022 Apr 22;7(8):e156341. doi: 10.1172/jci.insight.156341 (PMC9089784; doi:10.1172/jci.insight.156341)
Supplement: Supplemental data [file jciinsight-7-156341-s027.pdf]

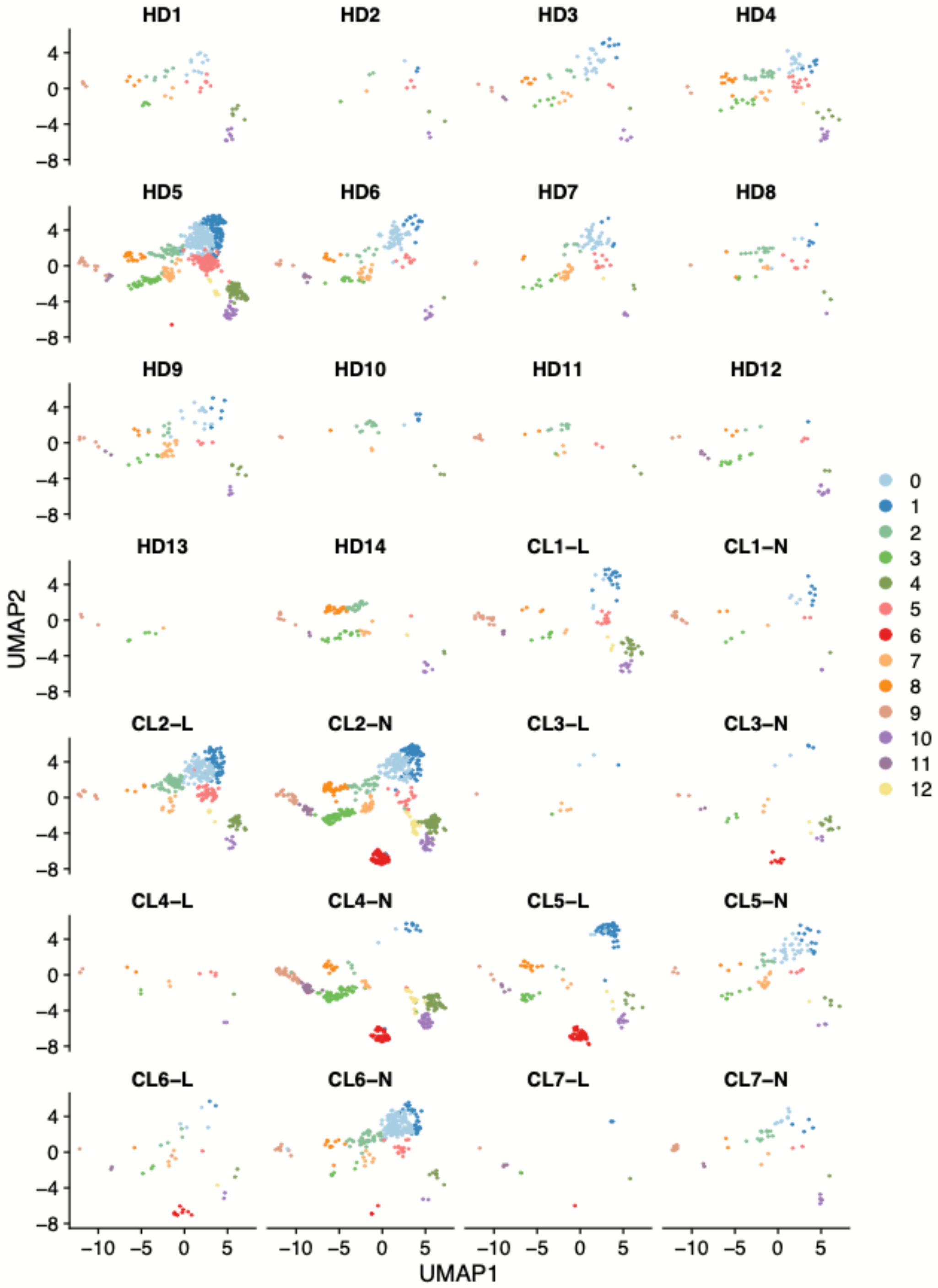

Supplementary Figure 1. UMAP plot locations of the cells from each sample.

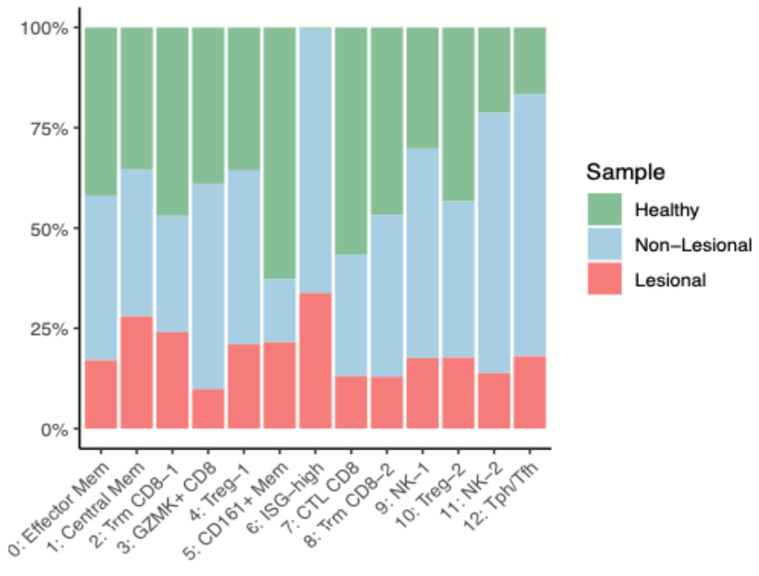

Supplementary Figure 2. Barplot of the healthy, non-lesional, and lesional components of each cluster.

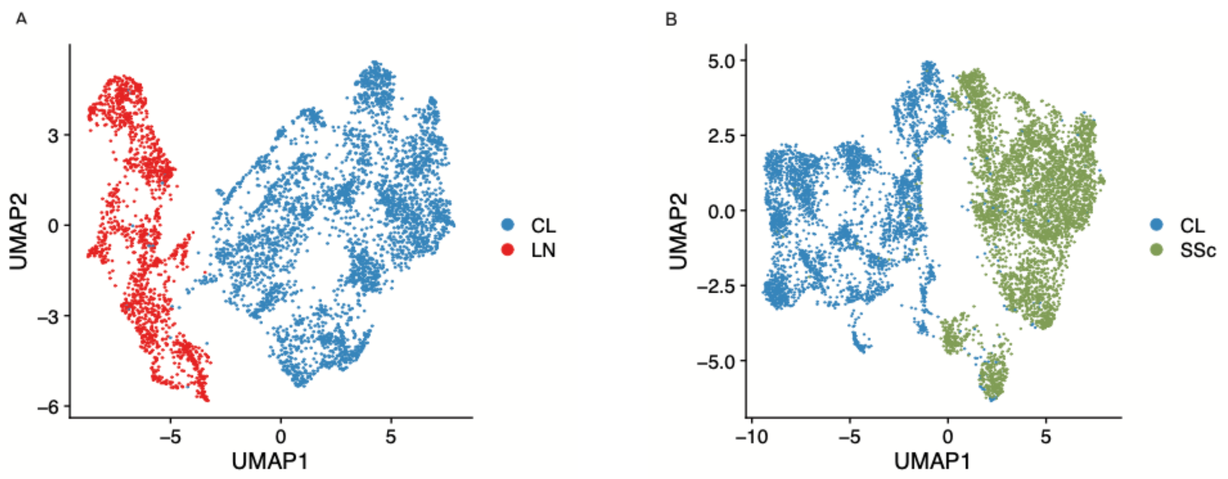

Supplementary Figure 3. A. UMAP plot of the merged cutaneous lupus (CL) and lupus nephritis (LN) datasets before CCA integration. **B.** UMAP plot of the merged CL and systemic sclerosis (SSc) datasets before CCA integration.

[See uploaded file]

Supplementary Table 1. Clinical characteristics of cutaneous lupus patients included in this study.

[See uploaded file]

Supplementary Table 2. Genes differentially expressed across T/NK clusters in skin biopsy samples.

[See uploaded file]

Supplementary Table 3. Signature gene lists for activation, cytotoxicity, exhaustion, and interferon response.
